# *Leishmania infantum*-Derived Glycoinositolphospholipids in the Immunodiagnosis of Subclinically Infected Dogs

**DOI:** 10.3389/fvets.2021.581148

**Published:** 2021-03-12

**Authors:** Julia Ramos Sampaio, Rodrigo Pedro Soares, Thiago Doria Barral, Gabriela Porfirio Passos, Maisa Santos Fonseca, Roberto Meyer, Stella Maria Barrouin-Melo, Ricardo Wagner Portela

**Affiliations:** ^1^Laboratório de Imunologia e Biologia Molecular, Instituto de Ciências da Saúde, Universidade Federal da Bahia, Salvador, Brazil; ^2^Instituto Rene Rachou, Fundação Oswaldo Cruz, Belo Horizonte, Brazil; ^3^Departamento de Anatomia, Patologia e Clínicas, Escola de Medicina Veterinária e Zootecnia, Universidade Federal da Bahia, Salvador, Brazil

**Keywords:** glycoconjugates, ELISA, *Leishmania infantum*, dogs, immunodiagnosis

## Abstract

Lipophosphoglycan (LPG), when used as an ELISA target, confers high specificity and sensitivity to the detection of *Leishmania infantum* antibodies in dogs. Glycoconjugates are economically viable because the yield is very high after extraction/purification. In addition, they are very stable, which allows their use in point-of-care testing without special storage conditions. During the glycoconjugate extraction, a glycoinositolphospholipid (GIPL)-enriched fraction is obtained in similar quantities as LPG. Since GIPLs can be extracted from the same parasite pellet as LPGs, this work aimed to evaluate the immune recognition of GIPLs by *Leishmania infantum*-infected dogs and its use for canine leishmaniasis (CanL) immunodiagnosis. Like LPG, GIPLs were recognized by sera from *L. infantum*-infected dogs, but with less sensitivity (83.8%). However, 80% (16/20) of subclinically infected dogs were detected as positive in the assay. Different from LPG, the GIPL-based assay achieved a lower specificity (73.7%) and cross-reactions occurred with *T. cruzi* and *L. braziliensis*-infected dogs. Although GIPLs exhibited a similar performance to LPG for subclinically *L. infantum*-infected dogs, the occurrence of cross-reactivities with other protozoa and a lower sensitivity hinders its use for an immunodiagnostic test. In places where those diseases do not co-exist such as in the Mediterranean region, its use for subclinically dogs could be an alternative.

## Introduction

Canine leishmaniasis (CanL) is a chronic zoonosis caused by *Leishmania infantum* (1). Domestic dogs (*Canis familiaris*) are the main sources of infection for vectors in urban areas representing a key element in the infection's epidemiology (2). Leishmaniasis is a spectrum of diseases and in the case of CanL caused by *L. infantum*, the clinical symptoms are variable, making it difficult to diagnose the infection (3). Dogs with high parasitic loads typically have more symptomatic and severe disease and are known to be more infectious to the sand fly vectors than resistant dogs (4). However, some susceptible dogs can have high parasitic loads without symptoms at the beginning of an active infection (3). Therefore, early diagnostic of CanL increases the chances for controlling the disease.

According to (5), an ideal diagnostic test includes an antigen that is able to confer high sensitivity, specificity and accuracy values, as well as having a high sensitivity in identifying subclinically infected dogs. This situation leads to the need for better diagnostic tests, mainly for subclinically infected dogs (6). Most studies on the search for better antigens focus on proteins/peptides identified by bioinformatics analyses (7–10). However, purifying those antigens can be expensive and involve complex methods. In this context, the search for non-protein antigens is an alternative to improve immunodiagnostics.

It was recently demonstrated that lipophosphoglycan (LPG) from *L. infantum* is an effective antigen to detect specific antibodies, especially in the initial stages of infection (11). Compared to proteins, glycoconjugates are very stable molecules. They can only be depolymerized by boiling at 100°C for 5 min in the presence of 0.02N hydrochloric acid (12). This characteristic is excellent for point-of-care diagnostic antigens. Both glycoinositolphospholipids (GIPLs) and LPGs are major *Leishmania* surface glycoconjugates. Both are immunomodulatory molecules and TLR2/4 agonists, being important in the parasite-host interaction (13, 14). One of the advantages of using *L. infantum* glycoconjugates (LPG and GIPL) is that the biochemical structures are known. Most (90%) of the *L. infantum* strains reported LPGs belonging to the type I family, whose repeat units are devoid of sidechains (15). *Leishmania infantum* GIPLs are also type I (mannosylated) with high similarity to those from *L. donovani* (13). LPGs are only present in the promastigote forms, while GIPLs are expressed at this stage and in the amastigote form in the vertebrate hosts. For this reason, this should increase the chances for glycoconjugates to induce a humoral immune response. A previous study reported the use of GIPLs for the diagnosis of ocular toxoplasmosis (16). However, no reports have evaluated the potential of GIPLs as antigens for CanL immunodiagnosis.

A distinguishing feature during the glycoconjugate purification protocol is the advantage of extracting two independent fractions containing similar amounts of LPG and GIPLs from the same parasite pellet. In this way, as part of a wider project on *Leishmania* glycoconjugates, we evaluated herein the role of GIPLs for CanL immunodiagnosis. This potentially increases the yield of antigen production from the same batch, providing a better economic viability for immunodiagnostics development.

## Materials and Methods

### Ethical Aspects

This study was approved by the Committee on Ethical Use of Experimental Animals of the Veterinary Medicine School of the Federal University of Bahia under the protocol number 023/2013.

### Sample Collection

Eighty *L. infantum*-infected dogs, as confirmed by PCR made with DNA purified from splenic aspirate samples (11), were clinically evaluated according to the classification system of (3, 17): G1—subclinically infected dogs (*n* = 20); G2—dogs with mild clinical disease (*n* = 24); G3—dogs with moderate clinical disease (*n* = 29); and G4—dogs presenting severe clinical disease (*n* = 7). Fifty-seven dogs from CanL non-endemic areas were used as negative controls. The use of reliable positive and negative samples is fundamental for the study of the recognition or not of the molecule by infected animals. For the cross reactivity tests, sera from dogs experimentally infected with *T. cruzi* in the acute (*n* = 10) and in the chronic phases (*n* = 10), and sera from dogs naturally infected with *L. braziliensis* (*n* = 11) were used. Results obtained herein were compared to the ones obtained at a previous study that developed an ELISA based on LPG to detect *L. infantum*-specific antibodies, which used the same serum samples that were tested herein; however, it was not possible to use all the serum samples from this previous study due to unavailability of some of them.

### Extraction and Purification of GIPLs

A *L. infantum* WHO reference strain Ba262 (MCAN/BR/89/Ba-262) isolated from a dog in Jacobina, Bahia state, Brazil was cultured for glycoconjugate extraction. GIPLs from stationary-phase promastigotes were extracted using chloroform:methanol:water (10:10:3) as previously reported (12). The solvent extract was dried by N_2_ evaporation and resuspended in 0.1 N acetic acid/0.1M NaCl. The suspension was applied to a column of phenyl-Sepharose (2 mL bed volume), equilibrated in the same buffer. GIPLs were eluted using solvent E (H_2_O/ethanol/diethyl ether/pyridine/NH_4_OH 15:15:5:1:0.017) and the concentration was measured by the phenol:sulphuric acid method (18).

### Standardization of GIPL-ELISA

The test was performed on 96-well flat adsorption polystyrene microplates (Perkin Elmer, Waltham, MA, USA), which were sensitized with GIPL antigens diluted in carbonate / bicarbonate buffer pH 9.6 at 100 μL / well and incubated at 4°C for 14 h. The plates were washed three times with PBS with 0.05% Tween 20 (PBST), blocked with PBST supplemented with 10% casein and incubated at 37°C for 2 h. After three washes with PBST, 100 μL of serum pool samples diluted in PBST 5% casein were added and incubated at 37°C for 1 h. Each serum sample was tested in duplicate. The plates were then washed with PBST four times and the anti-dog IgG peroxidase conjugated (Bethyl, Montgomery, TX, USA) diluted in PBST 5% casein was added to the plate, 100 μL per well, and incubated at 37°C for 1 h. After incubation, the plates were washed six times with PBST and 100 μL of a citrate buffer pH 5.3 added with 12 μL of H_2_O_2_ and 5 mg of orthophenylenediamine (OPD) (Sigma Aldrich, Saint Louis, MI, USA) was applied to each well. The reaction was stopped by adding 50μL of 4N H_2_SO_4_ in each well.

ELISAs were performed based on a checkerboard titration method following the one described by (11). First, different antigen concentrations (ranging from 0.125 to 2 μg/mL) and positive and negative sera pools dilutions (1:50, 1:100, 1:200, and 1:400) were used, with a fixed anti-canine IgG horseradish peroxidase antibody dilution (1:10,000). After the definition of the combined antigen concentration and the serum pool dilution that presented the higher positive pool optical density (OD)/negative pool OD ratio, a second checkerboard titration was performed, where these antigen concentrations and serum pool dilutions were tested with different secondary antibody dilutions (1:5,000, 1:10,000, 1:20,000, and 1:40,000). The positive and negative serum pools consisted of an equal quantity of ten negative or ten positive control serum samples. The combination of these different dilutions and concentrations that presented the higher positive/negative OD ratio was then chosen to individually test all the serum samples studied herein.

### Statistical Analysis

The results were interpreted as follows: truly positive samples were those presenting positive results by GIPL-ELISA and PCR, and the truly negative ones those with negative results at both assays. False positive samples were those from dogs living in CanL non-endemic areas scored positive by GIPL-ELISA and negative by PCR, while the false negative ones those scored negative by GIPL-ELISA but positive by PCR. The samples were considered as positive or negative at the GIPL-ELISA based on a cut-off calculated using the Receiver Operating Characteristic curve (ROC CURVE), obtained using a statistical software (SPSS v.12.0 software, IBM, USA), and the selection of the cut-off was based on the ROC curve point that presented the highest sum of sensitivity and specificity values. Thus, the sensitivity and specificity parameters were chosen based on the ROC curve, as previously described (11). The area under the curve was used to define the accuracy of the assay, and the negative and predictive values were calculated as previously described (19). The graphics were generated on the software GraphPad Prism 6.0 (Graph Prism Inc., San Diego, CA).

## Results

**Figure 1:**
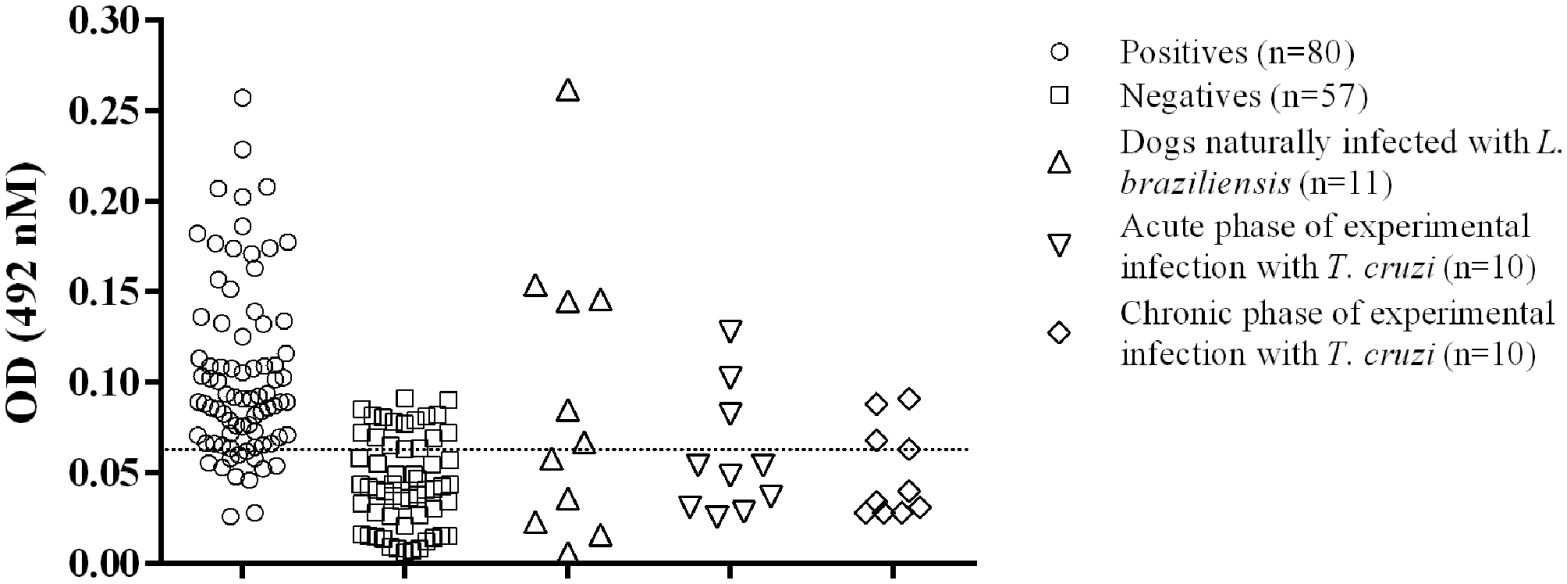
Distribution of the individual results of the positive and negative sera tested by GIPL-ELISA, and evaluation of cross reactivity in the GIPL-ELISA of serum from animals infected with other trypanosomatids. Regarding *L. infantum*, eighty positive and 57 negative serum samples were included in the study. Serum samples from dogs infected with *L. braziliensis* and *T. cruzi* (acute and chronic phases of the infection) were also tested. The line within the graphic represents the cut-off calculated from the ROC curve.

After the standardization of the indirect ELISA, we determined the best GIPL concentration, sera and conjugated antibody dilutions: 0.25 μg/mL, 1:200 and 1:10,000, respectively. OD results for each sample and control are shown in Figure 1.

The cut-off value, as calculated using the ROC Curve, was 0.064 for the GIPL-ELISA. Using this cut-off value, 67 of the 80 positive controls (83.75%) presented a positive result at the GIPL-ELISA, and 43 of the 57 negative controls (74%) presented a negative result. 16.25% (13/80) of the positive controls tested negative to *L. infantum* antibodies, and 24.6% (14/57) of the negative controls tested positive. The GIPL-ELISA, as performed, has 83.8% sensitivity and 73.7% specificity. Overall, the GIPL-ELISA presented 81 positive results and 56 negative results, leading to 82.7% of positive predictive value (PPV) and 76.8% of negative predictive value (NPV) (Table 1). This data was compared with LPG-ELISA data (right column of Table 1) (11). The accuracy (88.9%) was obtained using the area under the ROC curve (Supplementary Figure 1).

**Table 1:** GIPL-ELISA validation parameters.

**Parameters**	**GIPL-ELISA**	**LPG-ELISA (11)**
Number of samples tested	137	165
Number of positive sera	80	97
Number of negative sera	57	68
True positives	67	88
True negatives	43	67
False negatives	13	09
False positives	14	01
Cut-off	0.064	0.251
Sensitivity (%)	83.8	91.5
Specificity (%)	73.7	98.5
Accuracy (%)	88.9	99.7
Positive predictive value (PPV) (%)	82.7	98.9
Negative predictive value (NPV) (%)	76.8	89.3
Positive likelihood ratio	3.18	61

*CanL 80 positive and 57 negative control serum samples were tested. The parameters were calculated as previously defined (19)*.

Serum samples from dogs naturally *L. braziliensis*-infected showed positive reactions in 6/11 serum samples (54.5%) (Figure 1). Sera from *T. cruzi*-infected dogs showed positive reactions in 3/10 dogs in the acute phase of the infection (30%) and 3/10 in the chronic phase (30%) (Figure 1).

**Figure 2:**
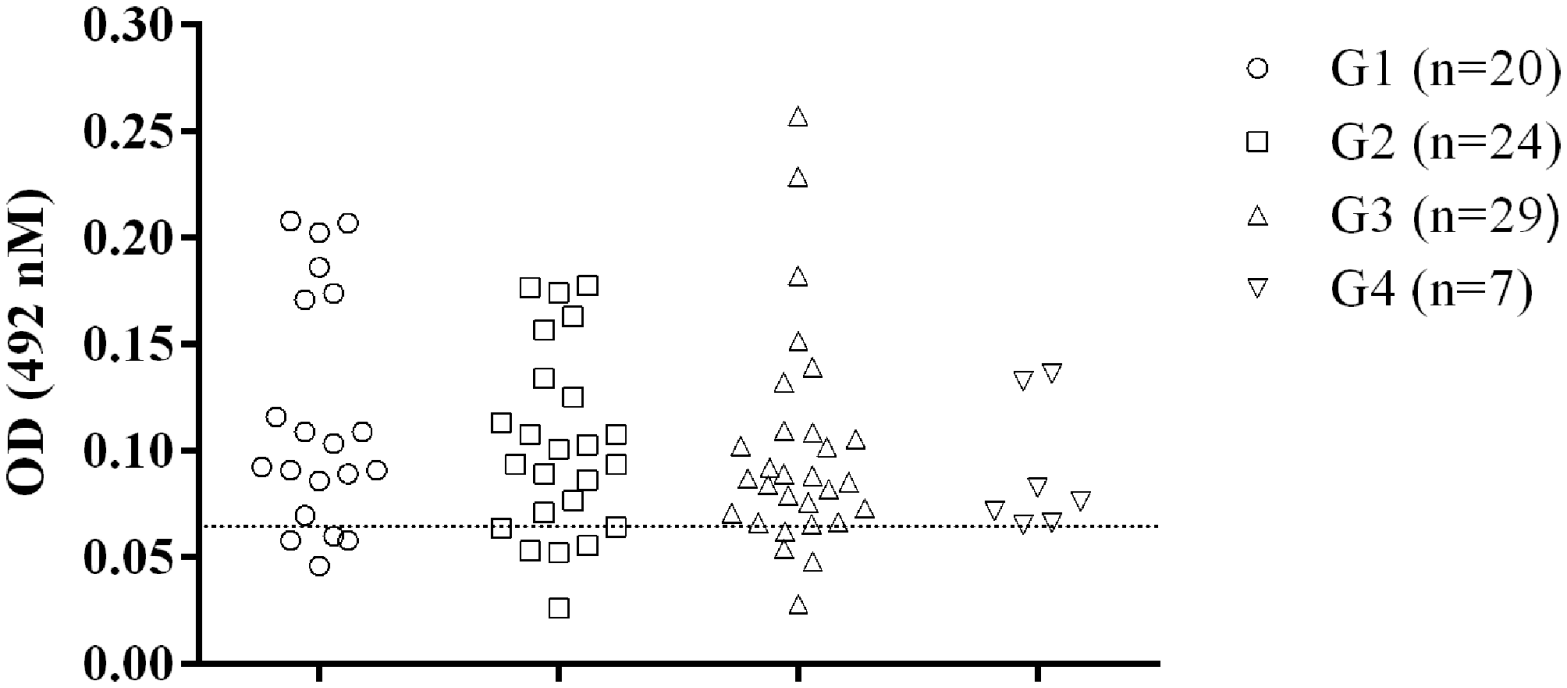
Recognition of GIPLs by sera from dogs at different Canine Visceral Leishmaniasis stages. The positive animals were classified according to the severity of *Leishmania* infection as previously described (11). The line within the graphic show the cut-off calculated from the ROC curve.

Of the dogs from the subclinically infected group (G1), 80% showed positive results in the assay with OD values above the cut-off. For the G2, G3 and G4 groups, it was found 79.2% (5/24), 86.2% (4/29), and 100% (7/7) of positive results, respectively (Figure 2).

## Discussion

In American and Mediterranean regions, dogs are the most important urban reservoirs of the *L. infantum* parasite (20). CanL immunodiagnosis is important because subclinically infected dogs, which appear healthy by physical examination and clinical pathology tests (3) may transmit the parasite before clinical manifestations appear. Due to subclinical cases, more advanced and sensitive techniques are required for early detection in order to control spread of the disease (1).

Our group previously reported that LPG is a promising antigen for CanL ELISA immunodiagnosis, detected 90% of subclinically infected dogs (11). *Toxoplasma gondii* GIPLs were previously reported to be excellent antigens for the diagnosis of human toxoplasmosis (16), and this fact made us investigate *L. infantum*-derived GIPLs as a candidate for CanL immunodiagnosis. This would made antigen obtention more economically viable, which is an important requirement during the development of a given immunodiagnostic test. It is important to point out that the LPGs and GIPLs used in our study were from a *L. infantum* dog-derived strain. GIPL-ELISA was able to detect 80% of the subclinically infected dogs. This significant sensitivity result for asymptomatic dogs was higher than those previously reported for immunoassays using *L. infantum* total lysate antigens and recombinant proteins (11, 21–23). Like LPGs, GIPLs exhibited false negative results in the G2 and G3 groups (11). However, unlike LPGs, GIPLs showed cross reaction with sera from *T. cruzi-* and *L. braziliensis*-infected dogs.

Although sera from *L. infantum*-infected dogs react to GIPLs, the optical densities measured were low, even when using higher amounts of antigen and lower dilution of samples, suggesting that there is a lower antigenic recognition of these molecules by canine IgGs. One possible explanation for the fact that LPG presented a better immune recognition than GIPL is the size of the molecules, since LPGs are bigger than GIPLs (24), and in this way can be more exposed in the parasite's surface.

GIPLs exhibited lower specificity and sensitivity values of 73.7 and 83.8% compared to 91.5 and 98.5% for LPGs. The GIPLs specificity results were probably lower due to cross-reactions in the serum samples of dogs infected by other protozoans. Cross reactivity is very common in diagnostic tests for CanL (25). Previous reports using an ELISA-based on the *L. infantum* LiHypA recombinant antigen that, despite obtaining high predictive values, showed cross reactivity with *Babesia canis* (26). In this study, the sensitivity values of the GIPL-ELISA when compared to the LPG ELISA was good (80% for GIPL and 90% for LPG), but its cross-reactivity hinders its use as an immunodiagnostic candidate especially in Latin American countries where CanL overlaps with Chagas disease. However, in Mediterranean countries where *T. cruzi* infection in dogs is rare or absent, GIPLs could be a promising alternative. Sera of dogs infected with other pathogens, including *Babesia* sp., *Ehrlichia* sp. and *Hepatozoon* sp., do not show cross reactivity with *L. infantum* LPG (11).

In this study, GIPLs were recognized by sera from 80% (16/20) of subclinically infected dogs. Previous studies using protein antigens reported subclinically infected dogs tested negative, but their assays had good sensitivity for dogs with clinically manifest CanL (21, 27). In addition, sera from subclinically infected dogs tested by an immunochromatographic assay (DPP-LVC rapid test, Bio-Manguinhos, Rio de Janeiro, Brazil) incorrectly tested as negative for infection (28). GIPLs are poor antigens to use for CanL immunodiagnosis, and the cross reactivity of sera from dogs infected by other parasites is a major obstacle to their use. However, the significant recognition of this molecule by subclinically infected dogs is an interesting result that may base further studies on the role of GIPLs in the host-parasite interaction in these animals, its use as a possible vaccine adjuvant and can be a choice of antigen to be used in *T. cruzi*-non endemic areas with the objective to detect asymptomatic dogs.

## Data Availability Statement

The original contributions presented in the study are included in the article/Supplementary Material, further inquiries can be directed to the corresponding author/s.

## Ethics Statement

The animal study was reviewed and approved by Committee on Ethical Use of Experimental Animals of the Veterinary Medicine School of the Federal University of Bahia.

## Author Contributions

All authors read and approved the final manuscript. JS, GP, TB, and MF: performed the experiments, interpretation of the data, and preparation of this manuscript. SB-M, RS, RM, and RP: funding obtaining, study design, technical review, and interpretation of the data.

## Conflict of Interest

The authors declare that the research was conducted in the absence of any commercial or financial relationships that could be construed as a potential conflict of interest.
