# Supplementary material for: Leishmania infantum-Derived Glycoinositolphospholipids in the Immunodiagnosis of Subclinically Infected Dogs
Source: Front Vet Sci. 2021 Mar 12;8:581148. doi: 10.3389/fvets.2021.581148 (PMC7994360; doi:10.3389/fvets.2021.581148)

Supplementary Figure 1. ROC curve of the positive and negative serum samples. Samples 80 positive and 57 negative) were tested using GIPL-ELISA. The curve was used for the determination of the cut-off value, the optical density with the optimal combined sensitivity and specificity.

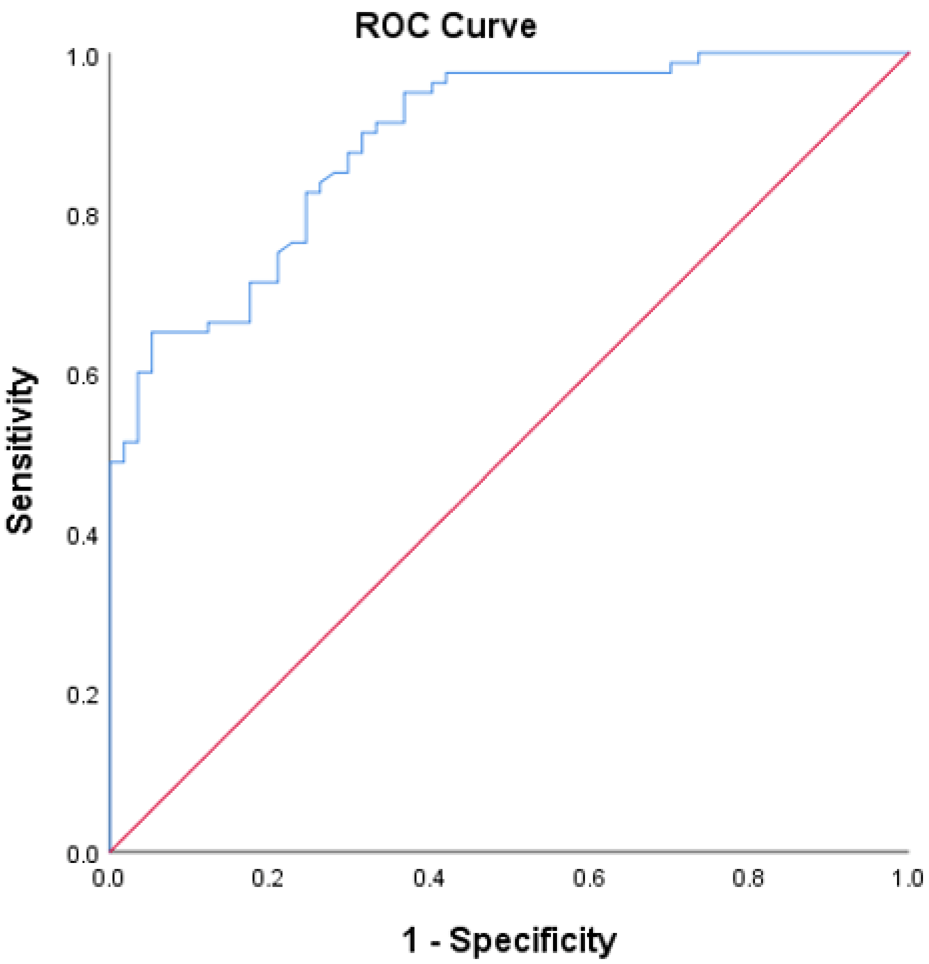

Supplement: Supplementary file 1 [file Data_Sheet_1.PDF]
